# Effects of Mindfulness Therapy on Breast Cancer Patients with Depression or Anxiety: A Systematic Review and Meta-analysis

**DOI:** 10.62641/aep.v53i2.1949

**Published:** 2025-03-05

**Authors:** Jingjing Yan, Fenhua Wang, Xianghua Yu

**Affiliations:** ^1^Department of Breast and Thyroid Ward, The Women’s and Children’s Hospital of Linping District Hangzhou, 311100 Hangzhou, Zhejiang, China; ^2^Department of Nursing, The Women’s and Children’s Hospital of Linping District Hangzhou, 311100 Hangzhou, Zhejiang, China

**Keywords:** mindfulness therapy, breast cancer, anxiety, depression

## Abstract

**Background:**

Breast cancer (BC) is the most common malignant tumor threatening women’s health globally, with rising incidence rates and significant psychological impacts, particularly in China, where the prevalence of depressive and anxious mood disorders among BC patients is notably higher than the global average. To evaluate the effect of mindfulness therapy on anxiety and depressive symptoms in breast cancer patients, as psychological distress significantly affects their quality of life and often persists despite medical treatment.

**Methods:**

A computerized search of Web of Science, PubMed, ScienceDirect, Cochrane Library, and Embase databases was conducted for randomized controlled trials involving the effects of positive thinking interventions on anxiety and depressive symptoms in breast cancer patients. The search was conducted from the time of database construction to December 2023. Two researchers completed literature screening, data extraction, and quality assessment, and then analyzed using RevMan 5.4 software.

**Results:**

A total of 15 studies involving 1823 patients were included. Meta-analysis results demonstrated that anxiety scores [mean difference (MD) = –0.67, 95% CI (–1.05, –0.29), *p* = 0.0005] and depression scores [MD = –2.26, 95% CI (–2.91, –1.61), *p* < 0.00001] were lower in the positive mindfulness intervention group than in the control group after the intervention, and the difference was statistically significant. Meanwhile, the follow-up time (>8 weeks vs ≤8 weeks) had little effect on the improvement of patients’ depression and anxiety scores. The distribution of studies was unsymmetrical, and there was a certain degree of publication bias.

**Conclusion:**

This study provides scientific evidence and practical guidance for psychological care in breast cancer patients, supporting the effectiveness of mindfulness-based interventions (excluding mindfulness-based cognitive therapy (MBCT)) in alleviating anxiety and depression. Future research should focus on high-quality randomized controlled trials to confirm and expand these findings and explore more effective intervention strategies.

## Introduction

Breast cancer (BC) is the most common malignant tumor that poses a threat to the physical and mental health of women worldwide. According to the latest global cancer statistics report, by 2020, the total number of newly diagnosed cancer cases in the world was total 19.3 million, and the number of BC among the new cancer cases was be as high as 2.26 million, accounting for 11.7% of the total number of cancer cases, and BC would formally replace lung cancer to become the world’s largest cancer [1]. The average incidence rate in Europe is 84/105 [2]. The lowest incidence occurs in the countries of Southeast Asia and Africa, where the standardized incidence rate does not exceed 25/105 [2]. The overall level of female breast cancer incidence in China was lower than that in other countries in Europe and the United States, but it was still on the rise relative to its prevalence trend [3].

As the coverage of breast cancer screening programs has become more widespread in China, the mortality rate of patients is gradually decreasing. However, many patients are still at the stage of being “scared of cancer”. Patients who are diagnosed for the first time will have adverse psychological reactions such as fear, anxiety and despair after learning about the disease. Later treatments, including surgery, radiotherapy and chemotherapy, can also lead to a negative psychological state of the patient, of which depression and anxiety disorders are the most common and serious [4]. The results of a meta-analysis study on the prevalence of depressive mood disorders in BC patients globally conducted by scholars such as Pilevarzadeh M *et al*. [5] and Hashemi SM *et al*. [6] showed that the prevalence of depressive and anxious mood disorders in female breast cancer patients globally were 32.2%, 41.9% respectively. And the prevalence of depression and anxiety mood disorders in female BC patients in China was 61.0% and 48.8%, respectively, which were higher than the global average [7]. Therefore, the selection of breast cancer patients as subjects in this study is meaningful.

Therapies such as surgery, chemotherapy and radiotherapy can treat the physical disease, but they cannot regulate the psychological state of the patients. Therefore, BC patients are often treated with complementary therapies centered on the construction of mindfulness, a therapy derived from Buddhist traditions that has been secularized and applied to multiple patient populations. Mindfulness is a way of awakening an individual’s inner focus on the present moment without judging it, primarily through the use of Eastern meditation [8]. Mindfulness-based interventions (MBIS) mainly include mindfulness-based stress reduction (MBSR), mindfulness-based cognitive therapy (MBCT), mindfulness-based art therapy (MBAT) and mindful awareness practices (MAPS) [9, 10]. MBSR is an 8-week structured program that includes sitting, meditation, yoga, and body relaxation. MBCT combines MBSR with cognitive-behavioral methods and focuses on mindfulness connection and psychoeducation. MBAT involves group art therapy combined with mindfulness meditation. MAPS includes positive thinking meditation, positive thinking walking, and psychoeducation for cancer survivors. Although existing studies have conducted systematic reviews and meta-analyses on the effectiveness of MBCT for breast cancer patients [11, 12], which have demonstrated significant effects in alleviating anxiety and depression, there are still some limitations. Firstly, existing systematic reviews primarily focus on MBCT, with relatively fewer studies on other forms of MBIS, such as MAPS and MBAT. Secondly, most existing studies concentrate on the effects of long-term interventions, with insufficient assessment of the effects of early interventions.

Therefore, this study aims to use meta-analysis methods to integrate and evaluate the effects of various forms of MBIS (except for MBCT) in alleviating anxiety and depression in breast cancer patients. By providing a comprehensive assessment of existing research, we hope to offer more detailed insights and provide scientific evidence and practical references for the clinical psychological care of breast cancer patients.

## Materials and Methods

### Literature Search

Computerized searches of Web of Science (https://www.webofscience.com/wos), PubMed (https://pubmed.ncbi.nlm.nih.gov), ScienceDirect (https://www.sciencedirect.com), Cochrane Library (https://www.cochranelibrary.com), and Embase (https://www.embase.com) databases were performed. The timeframe for the search was December 2023 when the database was constructed. Search strategies and search expressions: #1: breast cancer OR breast carcinoma OR breast neoplasm OR Paget’s disease; #2: mindfulness OR mindfulness meditation OR insight meditation OR mindfulness based OR stress reduction OR mindfulness based cognitive therapy; #3: anxiety OR anxiety state OR neurotic anxiety OR anxiety disorder; #4: depression OR depressive disorder OR depressive neurosis OR depressive syndrome OR neurotic depression; #5: #1 AND #2 AND #3 AND #4.

### Inclusion and Exclusion Criteria

Inclusion criteria: (1) Study type: randomized controlled trial (RCT); (2) Subjects: patients aged ≥18 years, diagnosed with breast cancer without other malignant tumors; (3) Intervention measures: the control group used conventional nursing measures or belonged to wait-list control; the intervention group used positive thinking intervention; (4) Endpoints: Anxiety and depression scores.

Exclusion criteria: (1) The ending indicators did not include anxiety and depression scores of the literature; (2) Duplicate published literature; (3) Literature published in the form of abstracts, reviews and case studies; (4) Literature reported in languages other than Chinese or English; (5) The full text and complete data could not be obtained by any means.

### Data Extraction and Bias Assessment

Two professionally trained researchers independently conducted literature search and screening in the above databases according to the established search strategy, inclusion and exclusion criteria. Firstly, the retrieved literature was screened by reading the title and abstract of the literature, and then the full text was read for a second screening and finally included in the literature. In case of disagreement, the decision was referred to a third-party arbitration panel. Information was extracted using a pre-made literature characterization form, which included the included studies, authors, year of publication, age, sample size, interventions, controls, and duration of follow-up. Two reviewers independently evaluated the quality of the included articles. The Cochrane Risk of bias tool was used to evaluate the quality of randomized controlled trials (RCTS). If the reviewers had any disagreement about the quality of the literature, the decision would be made after discussion with the third reviewer.

### Statistical Methods

The included studies were analyzed using RevMan 5.4 software (The Nordic Cochrane Centre, Copenhagen, Denmark). Heterogeneity was analyzed using Q-test combined with I^2^ value. If *p* > 0.10, I^2^ <50%, the heterogeneity between studies was considered acceptable and a fixed-effect model was used. If *p* ≤ 0.10 and I^2^ ≥50%, the heterogeneity among studies was considered large, and a random-effects model was used, and the heterogeneity was traced using sensitivity analysis and subgroup analysis. If the source of heterogeneity could not be determined, descriptive analysis was performed. Continuous variables were analyzed by using mean difference (MD) and calculating 95% Confidence interval (CI). PRISMA_2020_checklist in **Supplementary Material 1**.

## Results

### Results of Literature Search

**Fig. 1.**
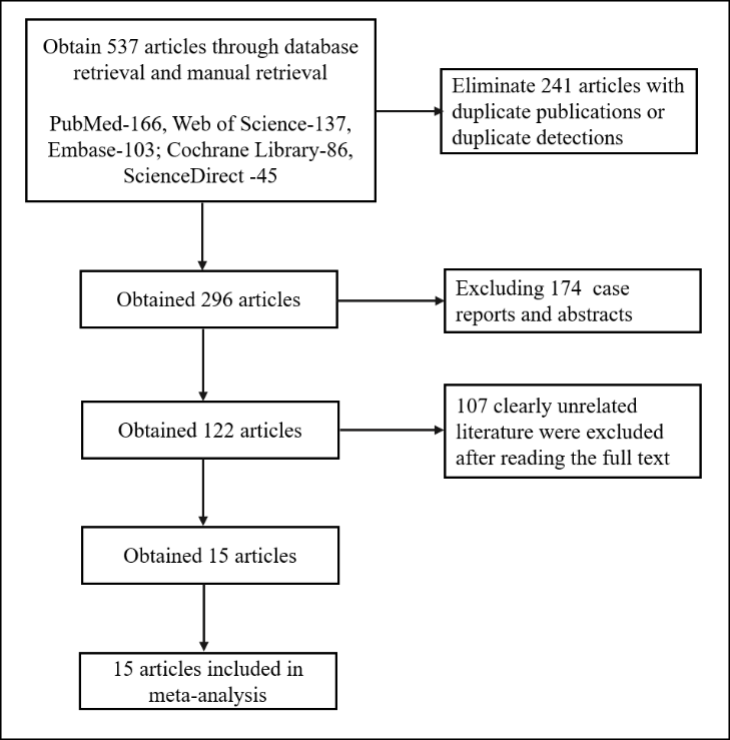
**Literature screening procedure and results**.

A total of 537 documents were retrieved, and duplicates in each database were excluded, and literature screening was performed according to the inclusion and exclusion criteria, and 15 documents were finally included, all of which were in English, and the process and results of literature screening are shown in Fig. 1.

### Literature Quality Bias Evaluation

**Fig. 2.**
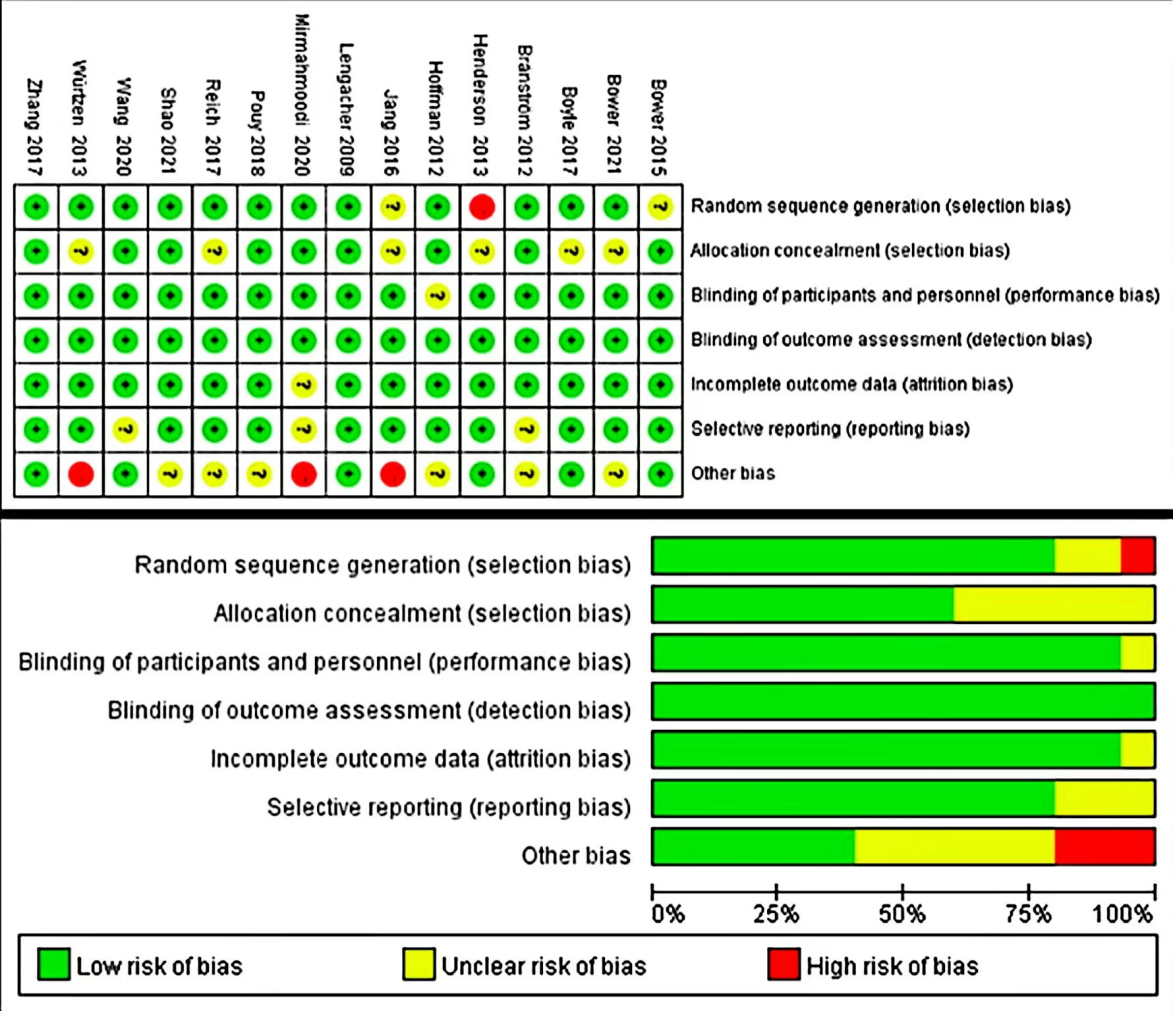
**Risk of bias assessment of the included literature**.

The quality of the included literature was evaluated according to the Cochrane Handbook evaluation criteria (Fig. 2). Of the 15 included studies, 7 were of grade A quality and 8 were of grade B quality, with credible results. 12 studies described random allocation methods, and 9 studies implemented hidden groups and used blinding of outcome evaluators. Some studies did not report concealed grouping and other biases, which may have resulted in some measurement bias and selectivity bias.

### Basic Characteristics

The 15 included literatures were published from 2009 to 2021, and all were RCT studies with a total of 1823 breast cancer patients (Table 1, Ref. [13, 14, 15, 16, 17, 18, 19, 20, 21, 22, 23, 24, 25, 26, 27]).

**Table 1.** **Basic characteristics of the included studies**.

Author	Year	Cases (intervention group/control group)	Age [M ± SD/M (range)]		Intervening measure		Follow-up (week)	Indicators
			Intervention group	Control group	Intervention group	Control group		
Bower *et al*. [13]	2015	39/32	46.1 (28.4–60)	47.7 (31.1–59.6)	MAPS	WLC	12	(1)
Bower *et al*. [14]	2021	85/81	44.5 ± 7.7	45.9 ± 5.6	MAPS	WLC	12	(1)
Boyle *et al*. [15]	2017	39/32	46 (28–60)	48 (31–60)	MAPS	WLC	12	(1)
Bränström *et al*. [16]	2012	32/39	–	–	MBSR	WLC	24	(1) (2)
Henderson *et al*. [17]	2013	53/58	–	–	MBSR	UC	16	(1) (2)
Hoffman *et al*. [18]	2012	114/115	49.0 ± 9.26	50.1 ± 9.14	MBSR	WLC	12	(1) (2)
Jang *et al*. [19]	2016	12/12	51.75 ± 5.32	51.42 ± 6.33	MBAT	UC	12	(1) (2)
Lengacher *et al*. [20]	2009	41/43	56.1 ± 9.1	58.0 ± 10.2	MBSR	UC	6	(1) (2)
Mirmahmoodi *et al*. [21]	2020	27/24	44.14 ± 11.9	45.64 ± 10.11	MBSR	UC	8	(1) (2)
Pouy *et al*. [22]	2018	35/35	52.12 ± 11.07	56.14 ± 11.04	MBSR	UC	8	(1) (2)
Reich *et al*. [23]	2017	159/152	58.0 ± 10.3	58.2 ± 9.5	MBSR	UC	6	(1) (2)
Shao *et al*. [24]	2021	72/72	40.3 ± 7.0	44.4 ± 8.2	MBIS	WLC	4	(1) (2)
Wang *et al*. [25]	2020	44/44	–	–	MBSR	UC	6	(1) (2)
Würtzen *et al*. [26]	2013	131/143	53.9 ± 10.09	54.39 ± 10.53	MBSR	UC	8	(1) (2)
Zhang *et al*. [27]	2017	28/30	48.67 ± 8.49	46.00 ± 5.12	MBSR	UC	8	(2)

SD, Standard Deviation; MAPS, mindful awareness practices; MBSR, mindfulness-based stress reduction; MBAT, mindfulness-based art therapy; MBIS, mindfulness-based interventions; WLC, wait-list control; UC, usual care; (1), Depression; (2), Anxiety.

### Meta Analysis of Depression

**Fig. 3.**
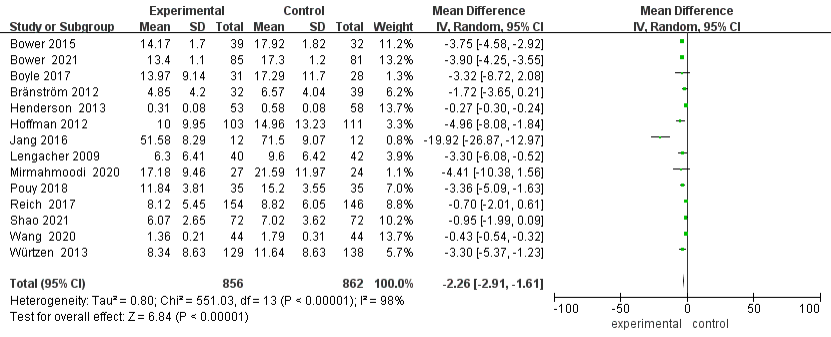
**Forest plot of depression levels**. CI, Confidence interval; SD, Standard Deviation.

Fifteen studies reported on patient depression (Fig. 3). After exclusion of lost patients from the studies of Boyle, Hoffman, Lengacher, Reich and Würtzen [15, 18, 20, 23, 26], 1718 patients were finally included. Heterogeneity between studies was high (*p* < 0.00001, I^2^ = 98%) and a random effects model was chosen. The results showed a statistically significant difference in depression scores between the two groups of patients after mindfulness intervention [MD = –2.26, 95% CI (–2.91, –1.61), *p* < 0.00001].

### Subgroup Analysis of Depression Levels

**Fig. 4.**
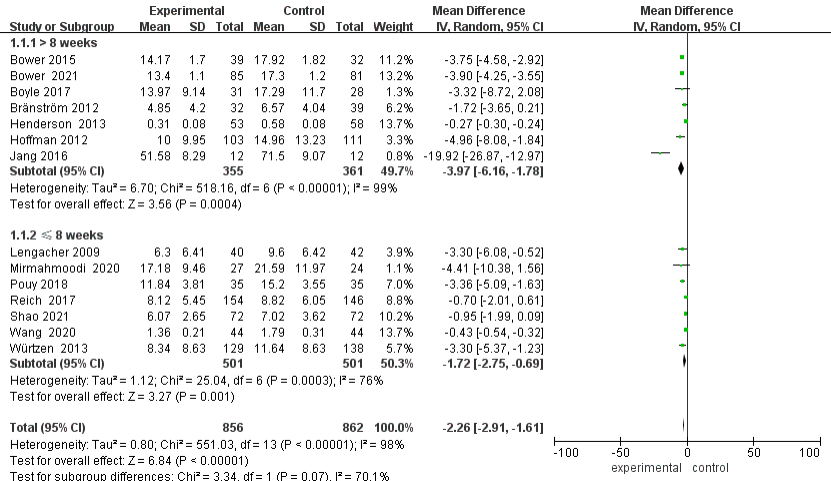
**Forest plot for depression subgroup analysis**. CI, Confidence interval; SD, Standard Deviation.

**Fig. 5.**
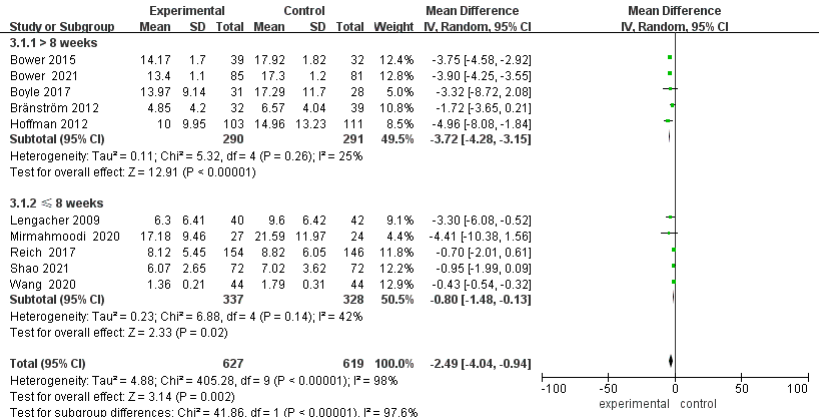
**Sensitivity analysis of depression levels**. CI, Confidence interval; SD, Standard Deviation.

Regardless of whether the length of follow-up was greater than 8 weeks, the depression scores of the intervention group were lower than those of the control group [≤8 weeks: MD = –1.72, 95% CI (–2.75, –0.69), *p* = 0.001; >8 weeks: MD = –3.97, 95% CI (–6.16, –1.78), *p* = 0.0004]. Sensitivity analyses were performed and studies were excluded on a case-by-case basis (Fig. 4). Heterogeneity was significantly reduced after excluding the Henderson *et al*. [17] and Jang *et al*. [19] study with a follow-up time >8 weeks (*p* = 0.26, I^2^ = 25%). Heterogeneity was also significantly reduced for follow-up times ≤8 weeks after excluding the study by Pouy *et al*. [22] and Würtzen *et al*. [26] (*p* = 0.14, I^2^ = 42%). When analyzing the reasons, the source of heterogeneity may be related to the different assessment scales. After excluding these studies, the results showed a statistically significant difference in post-intervention depression scores between the two groups of patients [MD = –2.49, 95% CI (–4.04, –0.94), *p* = 0.002], which is in the same direction as the previous results. The excluded study also showed a statistically significant difference between the two groups of patients comparing their depression levels after the intervention (*p* < 0.05), with a more stable result (Fig. 5).

### Meta Analysis of Anxiety

**Fig. 6.**
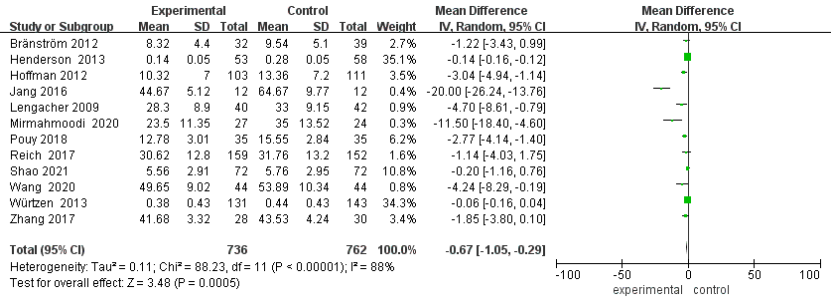
**Forest plot of anxiety levels**. CI, Confidence interval; SD, Standard Deviation.

Twelve studies reported patient anxiety status (Fig. 6). After excluding the lost patients from Hoffman *et al*. [18] and Lengacher *et al*.’s study [20], 1498 patients were finally included. Heterogeneity among studies was high (*p* < 0.00001, I^2^ = 88%) and a random effects model was chosen. The results showed a statistically significant difference in anxiety scores between the two groups of patients after the positive thinking intervention [MD = –0.67, 95% CI (–1.05, –0.29), *p* = 0.0005].

### Subgroup Analysis of Anxiety

**Fig. 7.**
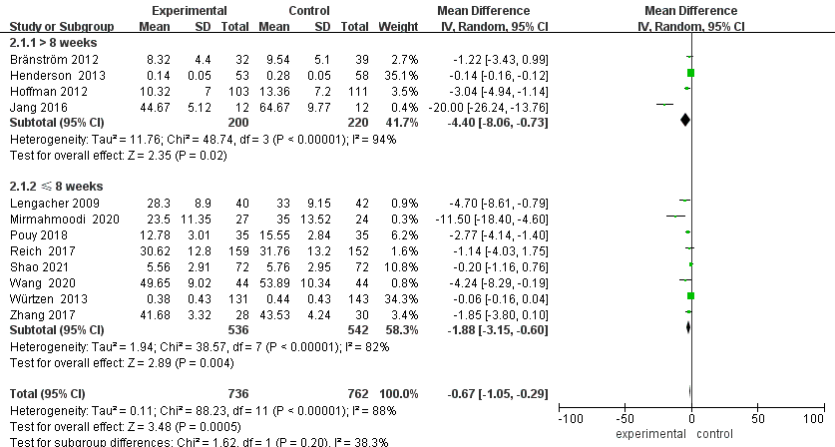
**Forest plot for anxiety subgroup analysis**. CI, Confidence interval; SD, Standard Deviation.

**Fig. 8.**
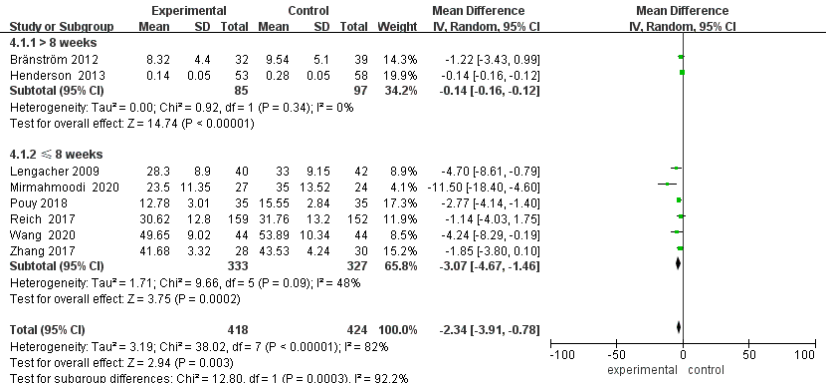
**Sensitivity analysis of anxiety levels**. CI, Confidence interval; SD, Standard Deviation.

Regardless of whether the length of follow-up was >8 weeks or not, the anxiety scores in the intervention group were lower than those in the control group [≤8 weeks: MD = –1.88, 95% CI (–3.15, –0.60), *p* = 0.004; >8 weeks: MD = –4.40, 95% CI (–8.06, –0.73), *p* = 0.02] (Fig. 7). Sensitivity analysis was performed by excluding studies one by one. Heterogeneity was significantly reduced with the exclusion of the Hoffman *et al*. [18] and Jang *et al*. [19] study with a follow-up time >8 weeks (*p* = 0.34, I^2^ = 0%). Heterogeneity was also significantly reduced for follow-up time ≤8 weeks after excluding Shao *et al*.’s study [24] (*p* = 0.09, I^2^ = 48%). When analyzing the reasons, the source of heterogeneity could be related to differences in assessment scales or interventions. After excluding these studies, the results showed a statistically significant difference in post-intervention anxiety scores between the two groups of patients [MD = –2.34, 95% CI (–3.91, –0.78), *p* = 0.003], which is in the same direction as the previous results. The excluded studies also showed a statistically significant difference in the comparison of anxiety levels between the two groups of patients after the intervention (*p* < 0.05), which is a stable and reliable result (Fig. 8).

### Funnel Plot

**Fig. 9.**
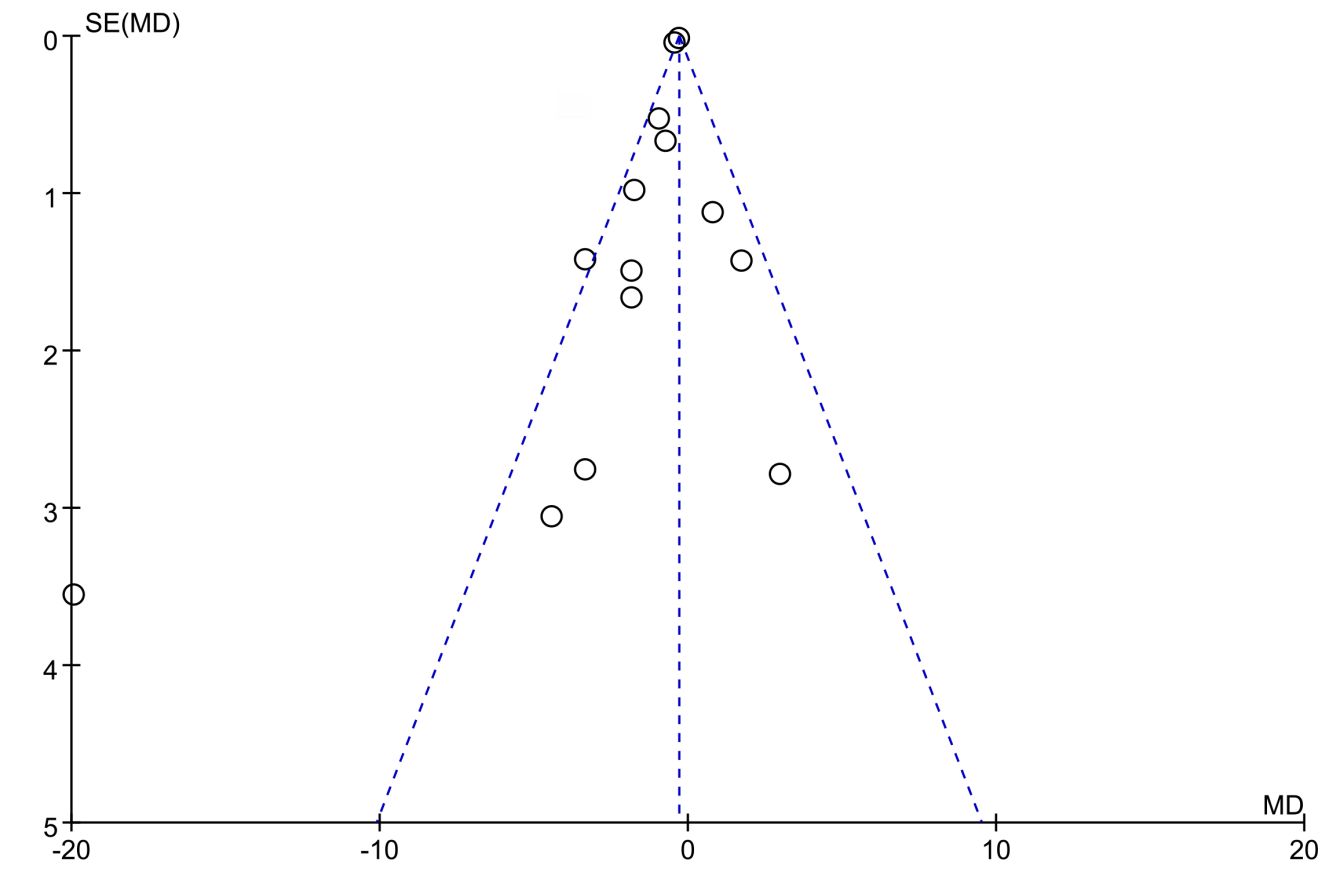
**Funnel plot of depression levels**. MD, mean difference; SE, standard error.

Publication bias was assessed visually using funnel plots. The funnel plot for the meta-analysis of depression levels is shown in Fig. 9. The funnel plot displayed an uneven distribution with three literature studies distributed on the right side, eight on the left side and four on the midline. The 15 papers didn’t have good left-right symmetry of funnel plot. Consequently, it can be inferred that this meta-analysis is subject to a certain degree of publication bias.

## Discussion

This systematic review and meta-analysis aimed to evaluate the effectiveness of various MBIS, including MBSR, MBAT, and MAPS, in alleviating anxiety and depression symptoms in breast cancer patients. Our findings indicate that these MBIS significantly reduce anxiety and depression symptoms in this patient population.

Existing research has primarily focused on MBCT, with comparatively less attention given to other forms of MBIS. Zainal *et al*.’s study [28] included only 2 RCTS, Cramer *et al*.’s study [29] did not compare pre- and post-intervention changes, and domestic studies have primarily examined the effects of MBSR alone, highlighting a gap in the comprehensive analysis of MBIS. By incorporating 15 relevant studies, our research addresses this gap by evaluating MBAT and MAPS, revealing their significant effects in alleviating depressive symptoms among breast cancer patients. Our subgroup analysis demonstrated that mindfulness interventions are effective in reducing depressive symptoms regardless of follow-up duration, underscoring the importance of early interventions in managing these symptoms. However, some limitations exist in the studies included. For example, one study found that the lack of significant long-term effects of MBIs on depressive symptoms might be related to patient dropout rates [30]. Our study observed high heterogeneity, likely due to variations in assessment tools and intervention methods. Excluding certain studies significantly reduced heterogeneity, suggesting that mindfulness interventions have a stable effect on alleviating depressive symptoms. Future research should focus on high-quality studies and consistent evaluation methods to improve the consistency and reliability of results.

Our analysis also demonstrated that mindfulness interventions are effective in reducing anxiety symptoms in breast cancer patients. Although most studies used MBSR, MBAT also showed significant effects in some studies, such as Jang *et al*. [19], which found notable improvements in anxiety symptoms with MBAT. Heterogeneity analysis indicated that differences in intervention effects might be related to variations in assessment tools and intervention methods. Future research should standardize assessment tools and compare different intervention methods to provide more accurate evaluations.

While this study provides a comprehensive evaluation of the effectiveness of mindfulness interventions for alleviating anxiety and depression symptoms in breast cancer patients, there are still some limitations. Firstly, the majority of included studies were in English, potentially overlooking relevant research in other languages. Secondly, the limited number of studies on MBAT and MAPS precluded a thorough comparison of these interventions. Lastly, factors influencing the severity of anxiety and depression symptoms, such as diagnosis time, cancer stage, and surgical methods, were not explored. However, this systematic review and meta-analysis evaluated the efficacy of various MBIs in alleviating anxiety and depressive symptoms in breast cancer patients, including MBSR, MBAT, and MAPS. This comprehensive assessment provides a more thorough understanding of the impact of different mindfulness interventions on the psychological health of breast cancer patients. Subgroup analyses indicated that mindfulness interventions were effective in reducing depressive symptoms regardless of follow-up duration, highlighting the importance of early intervention. Our study offers new insights into managing anxiety and depression in breast cancer patients. It identifies gaps in the existing literature, including the need for further research on the efficacy of MBAT and MAPS. Additionally, we observed high heterogeneity among the included studies, underscoring the need for standardized assessment tools and intervention methods. To enhance the quality and effectiveness of future research, we propose the following specific recommendations: (1) Include larger sample sizes to improve statistical power and explore the long-term effects of different mindfulness intervention strategies; (2) Employ standardized methods and tools during intervention implementation to reduce heterogeneity among studies and improve consistency and reliability of the results.

## Conclusion

This study provides scientific evidence and practical reference for the psychological care of breast cancer patients, supporting the effectiveness of MBIS in alleviating anxiety and depression symptoms. Our findings indicate that various MBIS, including MBSR, MBAT, and MAPS, are effective in reducing these symptoms. Future research should focus on conducting high-quality randomized controlled trials to verify and expand these findings, and to explore more effective intervention strategies.

## Availability of Data and Materials

The datasets used and/or analyzed during the current study were available from the corresponding author on reasonable request.

## Supplementary Material

Supplementary material associated with this article can be found, in the online version, at https://doi.org/10.62641/aep.v53i2.1949.
